# Age Characteristics of Patients With Type 2 Diabetic Foot Ulcers and Predictive Risk Factors for Lower Limb Amputation: A Population-Based Retrospective Study

**DOI:** 10.1155/jdr/2380337

**Published:** 2024-12-19

**Authors:** Yuanying Yao, Lei Chen, Yu Qian

**Affiliations:** ^1^Department of Medical Record Management, The First Affiliated Hospital of Wannan Medical College 241000, Wuhu, China; ^2^Department of Burn and Plastic Surgery, The First Affiliated Hospital of Wannan Medical College 241000, Wuhu, China; ^3^Department of Ultrasound Medicine, The First Affiliated Hospital of Wannan Medical College 241000, Wuhu, China

**Keywords:** age characteristics, diabetic foot ulcers, lower limb amputation, risk factors

## Abstract

**Background:** Limited data are available about the epidemiological characteristics and the risk factors for amputation, particularly in developing countries from Asia, especially in China.

**Objective:** We aim to investigate the age features of patients with Type 2 diabetic foot ulcers (DFUs) and analyze the critical influencing factors predicting lower extremity amputation and major amputation.

**Methods:** Data were retrieved from the electric medical record system to identify patients aged > 18 years with Type 2 DFU from January 1, 2017, to December 31, 2023. A logistic regression model was adopted to analyze the risk factors for amputation and major amputation.

**Results:** Nine hundred eighteen patients with Type 2 DFU were eligible for our study, 68.2% of whom were male. In patients with Type 2 diabetes in the hospitals we studied, the prevalence of Type 2 DFU was 1.07%. A majority of patients with DFU were in the 70–79 age group in the winter season, and deaths also peaked in this age group. A total of 38.8% of patients aged 50–59 years underwent amputation. Vascular CTA, complications, history of amputation, and infection sites were the important contributing factors in patients with DFU lower extremity amputation. History of amputation and hemoglobin were the main influencing factors of patients with major amputation resulting from DFU.

**Conclusion:** Most patients with DFU were in the age group of 50–59 years, but the majority of deaths occurred in the 70–79-year age group. Several factors influence the amputation, and those findings provide new insights into the relationship between the severity of narrowed blood vessels and the likelihood of amputation.

## 1. Introduction

The global prevalence of diabetes is predicted to increase to 578 million (10.2%) by 2030 and 700 million (10.9%) by 2045 [1], making it a leading chronic disease. Diabetic foot ulcers (DFUs) are one of the most common clinical complications in people with diabetes. It is characterized by the disruption of the foot skin and tissues, which is always invariably accompanied by peripheral neuropathy or arterial disease [2], that leads to increased risk factors for hospitalization and significant morbidity and mortality [3]. Global incidence of DFU within 1 year is around 6.3% [4], and in China, it is about 8.1% [5]. Moreover, the life expectancy of patients with DFU is similar to some common cancers, including colon and breast [6].

Research suggests that between 15% and 25% of individuals with diabetes may undergo DFU at some point during their lives [7]. The cumulative incidence of DFU is reported as 0.2%, 2.4%, and 5.8% for 12, 60, and 120 months, respectively [8]; of these, 40% to 60% of patients with DFU are treated with lower limb amputation [9]. Further, studies have indicated that diabetes patients face a significantly higher risk of lower extremity amputations, with rates 16 to 39 times higher than those without diabetes [10]. It is estimated that the mortality rate following amputation is alarmingly high with approximately 33% at 1 year [11, 12] and 42% to 44% of mortality after the onset of DFU, increasing to 68% to 79% in 5 years after amputation [13]. In addition, the incidence of mortality rate after major amputation (above the ankle) is 273.9 per 100,000 people, while after minor amputation, it is 113.4 per 100,000 (below the ankle) [14]. Furthermore, some studies found that amputation can lead to many negative consequences, including anxiety, depression, reduced quality of life, and the family distress, as well as increased healthcare costs [15]. In the United States, the average annual medical cost for a patient with diabetes is $8500; when it comes to amputation, the total cost increases from $43,800 to $66,215; and 77% of costs have occurred postamputation [16].

There are ample studies that compare DFU and amputation, including studies on risk factors, mortality rate, and treatment. Chamberlain et al. found that the national incidence of DFU in a retrospective cohort study of individuals with diabetes was 11.2 per 1000 people in Scotland [17]. However, the overall prevalence of DFU in Pakistan is 17.52%, and data revealed a higher frequency among females than males [18]. Diabetes-related amputation rates in Singapore remained stable, while the proportion of major amputations climbed from 63.6% in 2008 to 81.7% in 2017 [19]. Males, smokers, obesity [20], poor glucose control, low cholesterol levels, and elevated triglycerides [21] have been proven to lead to poor outcomes of amputation in people with diabetes. Moreover, some studies have suggested that intervention measures such as behavioral modification to reduce current smoking, heavy alcohol use, exercise [22], and a comprehensive therapeutic patient education [23] can prevent lower limb amputation. Although these researches are mainly focused on developed countries including England, Scotland, America, and Singapore, relative studies in developing countries from Asia are scarce. Different countries have disparate geographical environments, population characteristics, and social conditions, which have greatly influenced DFU and amputation, especially in China, where the situation is more complicated. Hence, it is essential to explore age features and epidemiological trends in patients with Type 2 DFU in Anhui, China, to identify prognostic influencing factors for amputations.

## 2. Methods

### 2.1. Study Design

A retrospective cohort investigation on the prevalence of DFU and amputation was initiated in Anhui Provence, China, by deriving the data from first of January, 2017, to 31st of December, 2023. The dataset comprised only hospitalized individuals, excluding those managed exclusively in the emergency or outpatient settings. Furthermore, the patients whose length of stay was less than 24 h were also excluded from the study, as this could potentially impact the accuracy of the results due to the incomplete information collected.

### 2.2. Study Population

The electronic medical record information system was used to extract the anonymous dataset, which is a demographic registry containing general demographic information and disease-related information for each patient during the period of hospitalization. Eligibility criteria were restricted to adults over the age of 18 years diagnosed with Type 2 DFU, and identified through International Statistical Classification of Diseases and Related Health Problems (ICD-10) Code E11.5. The following were deemed to be the exclusion criteria: (1) patients with serious mental illness and (2) patients with nonhealing refractory wounds due to previous trauma or foot surgery. If any critical patient data was unavailable within the digital medical records, compromising analytical validity, these cases were excluded from the study. Rigorous data cleansing protocols were enforced by the research team to maintain high-quality standards.

### 2.3. Procedures

The majority of demographic data, along with the majority of disease-related data, was gathered from the electronic medical record system, which primarily included information regarding gender and age, marital status, and laboratory data. Patients were divided into five age brackets: 18–49, 50–59, 60–69, 70–79, and ≧ 80 years. The onset of DFU was seasonally classified in which it occurred: spring (March–May), summer (June–August), autumn (September–November), and winter (December–February). In this study, the amount of DFU complications is referred to other conditions caused by diabetes other than DFU. A major amputation is defined as removal above the ankle, whereas a minor amputation is referred to as an excision of the limb below the ankle. Vascular computed tomographic angiography (CTA) is suggested in four categories: mild vascular stenosis, moderate vascular stenosis, severe vascular stenosis, and arterial occlusion. Two-hour postprandial C-peptide is referred to as 2 h-CP.

### 2.4. Statistical Analysis

Descriptive statistical analysis of data, such as percentages and counts, was conducted using SPSS Statistics V.20 software. All continuous variables were expressed as mean (standard deviation) and/or median (interquartile range), while the categorical variables were quantified as counts and percentages. Comparative analyses between groups for categorical variables were employed using either the chi-squared test or the Fisher test. Statistical significance was established at a two-tailed *p*-value threshold of < 0.05. To identify the influencing factors for lower limb amputation, major amputation, and minor amputation, a logistic regression analysis was conducted to meet the goal of the study, which involved the incorporation of variables with statistical significance (*p* < 0.05) derived from the univariate analysis.

## 3. Results

### 3.1. General Information of Study Population

A total of 918 patients with Type 2 DFU were eligible for our study out of a total of 85,872 patients with Type 2 diabetes. The study cohort consisted of 626 males (68.2%) and 292 females (31.8%). The prevalence of Type 2 DFU was 1.07% in all patients with Type 2 diabetes in the hospitals we studied. The average age was computed to be 61.86 with a standard deviation of 12.10 years, with the greatest number falling within the 50–59-year age group (*n* = 272, 29.6%), while the winter was observed as the peak season for DFU. Further, the median length of hospital stay was 16 days and the average duration of disease was 16.57 ± 12.51 years. Of the 918 patients with DFU, 565 patients had a toe infection, 51 had a heel infection, 79 had an acrotarsal infection, 50 had an ankle infection, 62 had a pelvic infection, and 111 had more than an infection. One hundred and seventy-eight patients had a lower limb amputation. One hundred and four patients had a history of amputation (11.3%); the average body mass index (BMI) was calculated to be 23.37 ± 3.07 kg/m^2^. The average values for albumin, hemoglobin, and white blood cells were determined to be 31.26 ± 6.76 g/L, 105.72 ± 22.63 g/L, and (31.26 ± 6.76)∗10^9^/L, respectively. A total of 98 patients with DFU had mild vascular stenosis (10.7%), 89 had moderate vascular stenosis (9.7%), 72 had severe vascular stenosis (7.8%), and 172 had arterial occlusion (18.7%). Patients with vascular stenosis and arterial occlusion accounted for 46.9% of people with DFU.

### 3.2. Characteristics of the Disease in Different Age Groups

The majority of patients with DFU were found to be in the age group of 50–59 years (*n* = 272, 29.6%), while the other age groups, that is, 70–79 years = 234 (25.5%), 60–69 years = 207 (22.5%), 18–49 years = 148 (16.1%), and ≧80 years = 57 (6.3%), showed relatively lesser count. Winter season was observed the maximum incidence of DFU, and patients aged 70–79 years accounted for the vast majority of the population among all the age groups in this season. Ten patients with DFU died during the hospitalization, of which four were aged 70–79 years. Among the patients with lower limb amputation, more than one-third were aged 50–59 years (*n* = 69, 38.8%), regardless of whether it was a major or minor amputation. The patients with DFU complicated with cardiovascular disease, cerebrovascular disease, and cardiovascular and cerebrovascular diseases were concentrated in the age group of 70–79 years old, with 34.4%, 35.5%, and 40.0%, respectively (Table 1).

### 3.3. Risk Factors for Amputation

#### 3.3.1. Univariate Analysis of Amputation

The univariate analysis revealed significant disparities (*p* < 0.05) in several factors between the patients undergoing lower limb amputation related to DFU and those not subjected to such procedures. These factors encompassed complications; locations of infection; the history of amputation; vascular CTA; levels of albumin, creatinine, and cholesterol; counts of white blood cells; hemoglobin levels; and interleukin-6 levels (Table 2). However, our analysis revealed that sex, marriage, season, comorbidity, disease duration, smoking, alcohol consumption, BMI, total protein, urea, triglycerides, high-density lipoprotein, low-density lipoprotein, glomerular filtration rate, red blood cells, HbA1c, 2 h-CP, and fasting C-peptide did not have a statistically significant effect (*p* > 0.05).

#### 3.3.2. Logistic Regression Analysis of Amputation

The dependent variable was found to be lower limb amputation; the independent variables were selected based on their statistical significance in the univariate analysis. Logistic regression analysis revealed that vascular CTA, complications, history of amputation, and infection sites were the contributing factors of patients with DFU lower limb amputation, among which arterial occlusion, no history of amputation, toe infection, and more than three complications were key risk factors for amputation (Table 3).

### 3.4. Risk Factors for Major and Minor Amputation

#### 3.4.1. Univariate Analysis of Major and Minor Amputation

The univariate analysis revealed statistically differences in patients with major and minor amputation in a number of variables, including marital status, seasons, history of amputation, albumin, urea, white blood cells, red blood cells, and hemoglobin (*p* < 0.05) (Table 4). But sex, age groups, comorbidities, complications, disease duration, smoking, alcohol consumption, infection sites, BMI, total protein, creatinine, glucose, cholesterol, triglycerides, high-density lipoprotein, low-density lipoprotein, glomerular filtration rate, HbA1c, 2 h-CP, interleukin-6, fasting C-peptide, and vascular CTA were not statistically significant in our results (*p* > 0.05).

#### 3.4.2. Logistic Regression Analysis of Major and Minor Amputation

The dependent variable was found to be major amputation, while the independent variables were selected based on their statistical significance in the univariate analysis. The findings of the logistic regression test suggested that history of amputation and hemoglobin were the influencing factors of patients with major amputation resulting from DFU, among which no history of amputation and higher hemoglobin were key risk factors for major amputation (Table 5).

## 4. Discussion

This study revealed a lower incidence rate of Type 2 DFU in Anhui, China, with a documented prevalence of merely 1.07% as compared to data obtained from Australia [24], Poland [25], and South Africa [26]. Nevertheless, it was approximately similar to the prevalence of DFU in Scotland [17], where the incidence of DFU was 1.12%. Our population-based study revealed a 19.4% amputation rate due to Type 2 DFU, which was lower than the results of study in Australia (29.8%) [27] and higher than in Malaysia (18.2%) [28]. This difference in incidence rate may contribute to disparities in geography and race, availability of medical facilities, the frequency of diabetes education, the sample size, and difference in research methods. Despite this, it is a fact that prevalence of Type 2 DFU and lower limb amputation in Anhui could provide some theoretical basis for the government to formulate preventive measures and diabetes education in the future.

The results showed that patients with DFU aged 50–59 years had a higher risk of amputation than those in other age groups, which was lower in the other studies at 70.8 years [27] and 64.8 years [29]. This may be because the wider age range in our study included DFU over 18 years of age. In addition, patients aged 50–59 years have less time for diabetes management education and may take their diabetes medication irregularly due to the huge economic and life burden before retirement, which increases the risk of amputation. Chung et al. [30] believed that temperature and humidity in winter delayed the recovery of diabetic wounds and increased the probability of amputation. The same case occurred in our study, especially in the age group of 70–79 years; patients with DFU have more comorbidities and underlying diseases in old age; and blood vessels become narrowed in winter, leading to a higher risk of death in this age group.

Furthermore, findings from this study demonstrated an association between various factors and lower limb amputation, with more than three complications that increase the likelihood of amputation, which corroborates the previous research in Ghana [31] and Africa [20]. The plausible explanation is that patients with diabetes and hyperglycemia have more complications, including diabetic peripheral neuropathy and diabetic peripheral vascular disease, resulting in impairing sensory function in the lower limb. Loss of sensation brought about non–wound healing, even the development of DFU, which eventually led to amputation. The findings indicate that more than three complications are predictors of amputation, which should be emphasized that the government should strengthen education and care for diabetes patients through timely detection and treatment of complications, so as to minimize the significant financial burden associated with amputations. However, smoking, alcohol consumption, and BMI were not risk factors for amputation in our study. The plausible explanation is that different areas have different culture and lifestyles; for example, compared to the people living in the north of China, people living in the south of China smoke less and have lesser alcohol consumption. From the medical records, we found that patients with DFU smoke an average of 10–20 cigarettes per day, while drinkers consume an average of 100 mL/day. Reduced smoking and alcohol consumption may not have had a significant effect on amputation. Due to the influence of geographical environment, the height and body size of people living in the south of China are smaller. The mean BMI of patients with DFU in our study was 23.37 ± 3.07 kg/m^2^, and the number of patients with normal BMI (185kg/m^2^BMI249kg/m^2^) was 670 (72.98%), so BMI did not have a statistically significant effect on amputation.

Recent research has suggested that the risk of a new DFU episode increases by 13-fold in patients who undergo a first amputation [32] and that the need for a second intervention for amputation increases by 21.2% [33]. Patients with diabetes with a history of amputation tend to experience poorer clinical outcomes, with an elevated risk of recurrent ulceration, amputation, and mortality versus those without such lesions [34]. However, our study revealed a significant distinction, such as the patients without a history of amputation exhibited an elevated likelihood of undergoing both major and minor amputations. This may be because individuals with a history of amputation receive proper diabetic foot care education from doctors and nurses after hospital admission, understand the importance of blood glucose control, and receive timely treatment. On the contrary, patients with DFU without a history of amputation who smoke and drink alcohol, do not treat open wounds timely, and lack knowledge about diabetic foot care are all at an increased risk of subsequent amputation.

Clinically, the degree of lower limb artery stenosis is the gold standard for assessing the feasibility of amputation, and the higher the degree of lower extremity arterial stenosis, the higher the risk of amputation [35]. In our study, 98.9% of individuals with lower extremity amputation underwent arterial stenosis, while 55.0% of amputation were contributed by patients with arterial occlusion. Severe stenosis or occlusion of the vascular brings about inadequate blood circulation, nutrients, and oxygen to the veins of the lower limb. This results in ulcers and gangrene if wounds fail to heal. Consequently, limb ischemia is important in poor wound healing and amputation. Currently, limited studies are available on the relationship between the degree of vascular stenosis and lower limb amputation. Thus, this study is of great significance for the early detection of vascular stenosis using vascular CTA, reducing the possibility of amputation and improving the prognosis.

Our study revealed that individuals with low hemoglobin had an elevated risk of requiring amputation, and there is a significantly lower hemoglobin level observed in patients undergoing major amputation than those undergoing minor amputation. Conversely, the blood hemoglobin level provides a certain degree of insight into the nutritional well-being of the patient, which is similar to the research showing that patients with poor nutrition have a higher the risk of amputation due to nutrition affecting amputation [36]. Additionally, patients with low hemoglobin have reduced erythropoietin, leading to ischemia and hypoxia, resulting in impaired renal function. Our findings indicated that patients with elevated levels of creatinine and urea levels had an increased chance of amputation, corroborating findings from other studies [37, 38]. Moreover, high creatinine and urea levels indicate impaired kidney function and chronic kidney disease. Renal complications in inpatients with diabetes indicate a narrowed or occlusion of peripheral blood vessels and a blockage of peripheral arterial blood circulation, increasing the possibility of lower extremity amputation. Theoretically, the higher the HbA1c level and the lower the glomerular filtration rate, the greater the risk of amputation; however, there was no statistical significance in our study, which may be necessary to further expand the sample size at a later stage to further investigate the association.

Despite its contribution, this study is not without limitations. First, the dataset employed in the study was obtained from the medical record system, which might contain coding errors by the coding clerk such as no type of diabetes, leading to a smaller sample size. Second, the present study focused exclusively on the prognostic elements linked to amputation in patients with Type 2 DFU from Anhui, which limits its ability to represent the epidemiological characteristics across China owing to different geographical features. Third, higher creatinine and urea levels were found to increase the risk of amputation. Still, we did not explore whether there were mediating variables, such as chronic kidney disease and diabetic liver disease. Future studies should gather more information on disease diagnosis to address this gap. Furthermore, this study solely explored the general demographic and clinical data as possible contributing factors to amputation; however, it did not investigate psychological factors, including anxiety, depression and frailty, family economic status, and type of medical insurance. Lastly, this study did not investigate the postamputation quality of life among patients after 3-year, 5-year, and 10-year survival rates, which needs further examination in subsequent follow-up studies.

## 5. Conclusion

The prevalence of Type 2 DFU was found to be 1.07% in all patients with diabetes in the hospitals we studied. A vast majority of patients with DFU were in the 70–79 age group in the winter season, and the deaths also peaked in this age group. The risk factors for lower limb amputation include vascular CTA, complications, history of amputation, and infection sites. In contrast, the contributing factors for major amputation are observed to be a history of amputation and hemoglobin. This study provides a theoretical basis for reducing the probability of amputation in clinical practice and highlights to the government to take some corrective measures to avoid lower limb amputation, including timely treatment of complications, the early detection of vascular stenosis through vascular CTA, adequate nutrition, and regular diabetes education. Furthermore, this study delivers pivotal insights into the correlation connecting the severity of narrowed blood vessels to the likelihood of undergoing lower extremity amputations, so that revascularization surgery can be used to reduce amputation and improve the prognosis.

## Figures and Tables

**Table 1:** Disease characteristics of patients with Type 2 DFU in different age groups (*n* = 918).

**Variables**	**18–49 years**	**50–59 years**	**60–69 years**	**70–79 years**	**≧ 80 years**	**χ** ^2^	**p** ** value**
Seasons						24.962	0.015
Spring	36 (13.6)	76 (28.8)	63 (23.9)	79 (29.9)	10 (3.8)		
Summer	25 (13.2)	73 (38.4)	39 (20.5)	43 (22.6)	10 (5.3)		
Autumn	34 (18.6)	59 (32.2)	40 (21.9)	38 (20.8)	12 (6.5)		
Winter	53 (18.9)	64 (22.8)	65 (23.1)	74 (26.3)	25 (8.9)		
States on discharge						42.344	< 0.001
Improved	109 (15.9)	184 (26.8)	159 (23.1)	192 (27.9)	43 (6.3)		
Cured	34 (20.5)	69 (41.6)	31 (18.7)	25 (15.1)	7 (4.1)		
Not cured	4 (7.3)	16 (29.1)	17 (30.9)	13 (23.6)	5 (9.1)		
Death	1 (10)	3 (30.0)	0 (0)	4 (40.0)	2 (20.0)		
Amputation						10.541	0.032
No	128 (17.3)	203 (27.4)	171 (23.1)	190 (25.7)	48 (6.5)		
Yes	20 (11.2)	69 (38.8)	36 (20.2)	44 (24.7)	9 (5.1)		
Level of amputation						1.750	0.782
Major amputation	3 (8.3)	14 (38.9)	6 (16.7)	10 (27.8)	3 (8.3)		
Minor amputation	17 (12.1)	55 (38.7)	30 (21.1)	34 (23.9)	6 (4.2)		
Comorbidities						114.628	< 0.001
None	120 (24.7)	168 (34.6)	102 (21.0)	82 (16.9)	14 (2.8)		
Cardiovascular disease	26 (7.5)	83 (24.0)	80 (23.1)	119 (34.4)	38 (11.0)		
Cerebrovascular disease	1 (3.2)	10 (32.3)	9 (29.0)	11 (35.5)	0 (0)		
Cardiovascular disease and cerebrovascular disease	1 (1.8)	11 (20.0)	16 (29.1)	22 (40.0)	5 (9.1)		

Abbreviation: DFU, diabetic foot ulcer.

**Table 2:** Comparison of relative information in patients with and without lower limb amputation due to Type 2 DFU (*n* = 178).

**Variables**	**Nonamputation**	**Amputation**	**All cases**	**χ** ^2^/**t**	**p** ** value**
Complications				172.201	< 0.001
None	395 (53.4)	7 (3.9)	402 (43.8)		
One	181 (24.5)	61 (34.3)	242 (26.4)		
Two	96 (13.0)	46 (25.8)	142 (15.5)		
More than three	68 (9.1)	64 (36.0)	132 (14.3)		
Infection sites				32.473	< 0.001
Toe	427 (57.7)	138 (77.5)	565 (61.5)		
Heel	46 (6.2)	5 (2.8)	51 (5.6)		
Acrotarsal	68 (9.2)	11 (6.2)	79 (8.6)		
Ankle	50 (6.8)	0 (0)	50 (5.4)		
Pelvic	58 (7.8)	4 (2.2)	62 (6.8)		
More than one	91 (12.3)	20 (11.3)	111 (12.1)		
History of amputation				13.278	< 0.001
No	670 (90.5)	144 (80.9)	814 (88.7)		
Yes	70 (9.5)	34 (19.1)	104 (11.3)		
Vascular CTA				346.600	< 0.001
None	485 (65.5)	2 (1.1)	487 (53.1)		
Mild vascular stenosis	85 (11.5)	13 (7.3)	98 (10.7)		
Moderate vascular stenosis	67 (9.1)	22 (12.4)	89 (9.7)		
Severe vascular stenosis	29 (3.9)	43 (24.2)	72 (7.8)		
Arterial occlusion	74 (10.0)	98 (55.0)	172 (18.7)		
Albumin^a^	31.78 ± 6.79	29.09 ± 6.19	31.26 ± 6.76	4.829	< 0.001
Creatinine^a^	136.94 ± 178.14	177.68 ± 240.40	144.84 ± 192.32	−2.546	0.011
Cholesterol^a^	3.75 ± 1.21	3.45 ± 1.20	3.69 ± 1.22	3.013	0.003
White blood cells^a^	8.70 ± 4.59	10.67 ± 6.34	9.08 ± 5.03	−4.76	< 0.001
Hemoglobin^a^	107.36 ± 22.76	98.88 ± 20.78	105.72 ± 22.63	4.540	0.001
Interleukin-6^a^	63.19 ± 97.49	85.87 ± 132.85	67.59 ± 105.58	−2.581	0.010

Abbreviation: DFU, diabetic foot ulcer.

^a^The units for albumin, creatinine, cholesterol, white blood cells, hemoglobin, and interleukin-6 are grams per liter, micromoles per liter, millimoles per liter, 10^9^/L, grams per liter, and picograms per milliliter, respectively.

**Table 3:** Logistic regression model examining the influence factors of lower limb amputation among patients with Type 2 DFU (*n* = 918).

**Variables**	**B**	**Standard error**	**Wals**	**Unadjusted**		**Adjusted**	
				**OR (95% CI)**	**p** ** value**	**OR (95% CI)**	**p** ** value**
Complications	−3.031	0.891	11.570	0.064 (2.272–3.172)	< 0.001	0.048 (1.005–1.115)	0.001
Infection sites	−0.204	0.061	11.242	0.347 (0.790–0.926)	< 0.001	0.816 (0.724–0.919)	0.001
History of amputation	1.050	0.365	8.288	0.215 (1.445–3.535)	< 0.001	2.857 (1.398–5.839)	0.004
Vascular CTA	1.172	0.095	151.645	2.904 (2.513–3.357)	< 0.001	3.228 (2.679–3.890)	< 0.001

*Note:* Weight cases: complications (none = 0, one = 1, two = 2, and more than three = 3); infection sites (toe = 1, heel = 2, acrotarsal = 3, ankle = 4, pelvic = 5, and more than one = 6); history of amputation (no = 0, yes = 1); and vascular CTA (none = 0, mild vascular stenosis = 1, moderate vascular stenosis = 2, severe vascular stenosis = 3, and arterial occlusion = 4).

Abbreviations: CI, confidence interval; CTA, computed tomographic angiography; DFU, diabetic foot ulcer; OR, odds ratio.

**Table 4:** Comparison of relative information in patients with major amputation and minor amputation due to DFU (*n* = 178).

**Variables**	**Major amputation**	**Minor amputation**	**All cases**	**χ** ^2^/**t**	**p** ** value**
Marriage				6.256	0.044
Spinsterhood	4 (57.1)	3 (42.9)	7 (100)		
Married	31 (18.6)	136 (81.4)	167 (100)		
Divorced	1 (25.0)	3 (75.0)	4 (100)		
Seasons				12.710	0.005
Spring	5 (10.2)	44 (89.8)	49 (100)		
Summer	17 (37.8)	28 (62.2)	45 (100)		
Autumn	5 (13.5)	32 (86.5)	37 (100)		
Winter	9 (19.1)	38 (80.9)	47 (100)		
History of amputation				5.915	0.015
No	24 (16.7)	120 (83.3)	144 (100)		
Yes	12 (35.3)	22 (64.7)	34 (100)		
Albumin^a^	24.85 ± 6.32	30.16 ± 5.69	29.09 ± 6.19	−4.891	< 0.001
Urea^a^	11.09 ± 8.95	8.55 ± 5.76	3.43 ± 0.67	2.083	0.039
White blood cells^a^	19.94 ± 7.57	9.59 ± 5.51	10.67 ± 6.34	4.800	< 0.001
Red blood cells^a^	3.13 ± 0.78	3.50 ± 0.61	3.43 ± 0.67	−3.059	0.003
Hemoglobin^a^	87.33 ± 18.60	101.80 ± 20.34	98.88 ± 20.78	−3.877	< 0.001

Abbreviation: DFU, diabetic foot ulcer.

^a^The units for albumin, urea, white blood cells, red blood cells, and hemoglobin are grams per liter, millimoles per liter, 10^9^/L, 10^12^/L, and grams per liter, respectively.

**Table 5:** Logistic regression model examining the influence factors of major amputation and minor amputation among patients with Type 2 DFU (*n* = 178).

**Variables**	**B**	**Standard error**	**Wals**	**Unadjusted**		**Adjusted**	
				**OR (95% CI)**	**p** ** value**	**OR (95% CI)**	**p** ** value**
History of amputation	−0.373	1.100	0.115	5 (0.160–0.840)	< 0.001	0.689 (0.815–0.925)	0.735
Hemoglobin^a^	0.011	0.011	11.484	1.042 (1.019–1.065)	< 0.001	1.040 (1.016–1.063)	0.001

*Note:* Weight cases: history of amputation (no = 0, yes = 1).

Abbreviations: CI, confidence interval; CTA, computed tomographic angiography; DFU, diabetic foot ulcer; OR, odds ratio.

^a^The unit for hemoglobin is grams per liter.

## Data Availability

The data that support the findings of this study are available from the corresponding authors upon reasonable request.
